# Patients’ knowledge, attitude, and practice toward stroke rehabilitation: a web-based cross-sectional study

**DOI:** 10.3389/fpubh.2025.1593802

**Published:** 2025-08-07

**Authors:** Xiaoling Qian, Haixia Wang, Xiaoyan Wang, Tingting Wu, Hongmei Han, Xia Bu, Fengling Teng

**Affiliations:** ^1^Department of Neurology, Lanzhou University Second Hospital, Lanzhou, China; ^2^Department of Cardiology, Lanzhou University Second Hospital, Lanzhou, China; ^3^Department of Neurosurgery, Lanzhou University Second Hospital, Lanzhou, China; ^4^Department of Technology, Lanzhou University First Hospital, Lanzhou, China

**Keywords:** knowledge, attitude and practice, stroke, rehabilitation, structural equation model, cross-sectional study

## Abstract

**Objective:**

The objective of this research was to comprehensively assess how well patients understand stroke rehabilitation, their perceptions of it, and their inclination to participate.

**Methods:**

This web-based cross-sectional study was conducted between February and June 2023 at the Second Hospital of Lanzhou University, using a self-administered questionnaire.

**Results:**

A total of 497 valid questionnaires were enrolled, including 342 (68.81%) males. The mean score of knowledge, attitude and practice was 11.79 ± 0.63 (possible range: 0–12), 36.06 ± 2.55 (possible range: 12–60), and 58.24 ± 5.08 (possible range: 14–70), respectively. The results demonstrated that knowledge has a positive and significant direct effect on attitudes (*β* = 0.249, *p* < 0.001), and attitudes had direct effects on practice (*β* = 0.443, *p* < 0.001). Knowledge had direct (*β* = 0.124, *p* = 0.002) and indirect effects (*β* = 0.111, *p* < 0.001) on practice.

**Conclusion:**

Stroke patients had sufficient knowledge, unfavorable attitude and positive practice toward stroke and rehabilitation training. This study showed that addressing and enhancing individuals’ attitudes could be a key strategy in promoting more positive and effective practice toward stroke and rehabilitation training among stroke patients.

## Introduction

Stroke results from the sudden rupture or blockage of cerebral blood vessels, causing brain damage and functional impairment. Its high incidence, mortality, and disability rates establish it as a significant health concern in China. Stroke’s increasing incidence, with over 2 million new cases annually, coupled with multiple risk factors, underscores its substantial burden (1, 2). The aftermath of stroke extends beyond physical effects, severely impacting psychological well-being and life quality. Survivors grapple with limb dysfunction, disrupting daily life and overall well-being. While current treatments aim to minimize damage, rehabilitation remains challenging, especially for those with physical or mental impairments (3, 4). Despite advancements in medical care, stroke imposes a significant burden on individuals, families, and healthcare systems worldwide. Survivors often experience long-term disability, reduced quality of life, and a high risk of recurrent stroke, which collectively contribute to substantial healthcare costs and loss of productivity.

Restorative therapies following a stroke are essential for helping survivors regain autonomy and enhance their overall life satisfaction. It enables survivors to regain motor function, improve cognitive and communication abilities, and adapt to daily activities, thereby reducing the psychological and social burdens associated with stroke. Many survivors grapple with functional impairments affecting limb control, language abilities, and cognitive functions (5–7). The aftermath of a stroke often triggers feelings of helplessness and poses significant challenges in adhering to rehabilitation, detrimentally affecting its effectiveness (8). Such challenges frequently lead individuals to adopt negative coping strategies, which hinder the recovery process. A lack of educational background further exacerbates emotional distress, impeding treatment adherence, reinforcing the need for comprehensive stroke treatments that encompass psychosocial aspects. In an evolving medical landscape, prioritizing patients’ quality of life gains prominence. Stroke rehabilitation transcends physical function to encompass psychological and social dimensions, ultimately enhancing overall well-being. Modern stroke care integrates early and sustained interventions, aiming to maximize recovery across domains and improve quality of life (9, 10). A positive Knowledge, Attitude, and Practice framework is instrumental in supporting stroke rehabilitation. Patients with adequate knowledge are more likely to understand the importance of timely interventions and adhere to prescribed rehabilitation regimens, while positive attitudes foster motivation and resilience during recovery.

The Knowledge-Attitude-Practice (KAP) framework offers a systematic approach for probing individuals’ understanding of health concepts, their mindset toward treatment, and their behavioral intentions. While previous studies have primarily focused on stroke awareness, acute ischemic stroke management, and prediction models for upper limb function recovery (11–13), few have explored the mediating role of attitudes in shaping patients’ practice to participate in rehabilitation. Most existing literature emphasizes either knowledge levels (11, 13) or behavioral outcomes (14, 15), often overlooking how attitudes may bridge the gap between understanding and action. By demonstrating that attitudes significantly mediate the effect of knowledge on practice, our study addresses this critical gap and contributes novel insights into the psychosocial dynamics of stroke rehabilitation engagement. This emphasizes a distinct gap in the academic landscape, signaling the potential value of exploring this uncharted territory. By focusing on this specific facet, the current study aims to contribute to a more comprehensive understanding of stroke rehabilitation, ultimately aiming to enrich the existing body of knowledge in the field. Therefore, this study aimed to investigate the KAP toward rehabilitation training among stroke patients.

## Methods

### Study design and participants

Between February and June 2023, a snapshot study was executed at a high-level medical center in Lanzhou, enrolling patients with stroke rehabilitation within the neurology and neurosurgery departments. The inclusion criteria were: (1) aged 18 or older; (2) patients adhering to the “Chinese guidelines for diagnosis and treatment of acute ischemic stroke (2018)” (16) diagnostic criteria with a definite onset date and confirmed diagnosis by CT or MRI; (3) mentally alert, cognitively sound for verbal and written communication; (4) willingly engaged in the questionnaire. The exclusion criteria were: (1) with profound impairments in vision, hearing, language, or cognition affecting cooperation (specifically, patients with Glasgow Coma Scale score <15, severe aphasia, or dementia); (2) with clinically significant missing data. Ethical clearance for the study was obtained from the institutional review board of Lanzhou University Second Hospital. The purpose and significance of the research were explained to patients and their families, and informed consent was obtained through signed consent forms.

### Questionnaire

Following guidelines (14, 17–19), a questionnaire was constructed spanning four dimensions. This questionnaire underwent iterative refinement with input from eight experts in neurology, neurosurgery, rehabilitation, and cerebrovascular diseases. Redundant questions were eliminated, and ambiguous items were clarified. Before official distribution, a pilot test was conducted with 52 participants yielded a satisfactory Cronbach’s *α* of 0.772 for internal consistency. The final version encompassed the following domains: (1) Demographic information, which included variables such as age, gender, residency type, education level, occupation, income, and other pertinent details. (2) The knowledge dimension consisted of 12 questions, with each correct response receiving a score of 1 point, while incorrect or unclear responses were assigned a score of 0. (3) The attitude dimension comprised 12 items evaluated on a five-point Likert scale, ranging from extremely positive (5 points) to extremely negative (1 point). (4) The practice dimension included 14 items, also rated on a five-point Likert scale, with responses ranging from always (5 points) to never (1 point). Scores for knowledge, attitude, and practice that met or exceeded 70% of the theoretical maximum were classified as adequate knowledge, positive attitude, and proactive practice, respectively (20).

For distribution, the questionnaire was uploaded to the Sojump website^1^ to create a QR code for the electronic questionnaires. Participants accessed the survey by scanning the QR code, with mandatory responses to ensure data completeness. Given the high mobile internet penetration in China, including among older adults, most participants were able to complete the online questionnaire independently. For participants with limited digital literacy, especially older adult or low-income individuals, trained nurses provided assistance. In cases where participants were unable to operate mobile devices, the nurse would read each questionnaire item aloud and complete the form on the participant’s behalf based on their responses. This approach helped reduce potential exclusion due to technical barriers. The research team reviewed each questionnaire for consistency and clarity.

### Statistical analysis

Statistical analysis was conducted using SPSS 26.0 (IBM Corp., Armonk, N.Y., United States). Quantitative variables were described using mean ± standard deviation (SD), and between-group comparisons were performed using t-tests or analysis of variance (ANOVA). Categorical variables were presented as *n* (%). The study employed structural equation model (SEM) to assess the hypotheses that knowledge directly effects attitudes and practice, and attitudes directly effects practice. All statistical tests were two-tailed, with significance set at *p* < 0.05. To determine the variables included in the multivariate logistic regression, a forward selection method was used. Independent variables that were significantly associated with practice scores in the univariate analysis (*p* < 0.05) were entered into the multivariate model. Multicollinearity diagnostics were performed, and all included variables showed acceptable tolerance values (>0.1) and Variance Inflation Factor (VIF) values < 1.2, indicating no evidence of collinearity.

## Results

### Demographic characteristics

Initially, a total of 502 questionnaires were gathered, among them, one is under 18 years old and two did not provide signed informed consent, and two questionnaires were blank. The final dataset consisted of 497 cases, resulting in an effective recovery rate of 99.00%. Among them, 342 (68.81%) were male, the mean age was (63.59 ± 12.32) years, and 235 patients (47.28%) resided in non-urban areas. In terms of occupational status, 204 patients (41.05%) were categorized as retired. Moreover, 129 patients (25.96%) reported an income of less than 2000 CNY. The marital status distribution revealed a significant majority, with 481 patients (96.78%) being married. In terms of health-related characteristics, 344 patients (69.22%) reported an illness duration of less than 1 month (Table 1).

**Table 1:** Demographic characteristics.

Variables	*N* (%)	Knowledge, mean ± SD	*p*	Attitude, mean ± SD	*p*	Practice, mean ± SD	*p*
Total score	*N* = 497	11.79 ± 0.63		36.06 ± 2.55		58.24 ± 5.08	
Gender			0.077		0.020		0.011
Male	342 (68.81)	11.81 ± 0.60		36.23 ± 2.50		58.58 ± 5.09	
Female	155 (31.19)	11.74 ± 0.69		35.69 ± 2.64		57.48 ± 4.99	
Age, years			0.539		0.881		0.518
<55	113 (22.74)	11.74 ± 0.67		36.25 ± 2.32		58.53 ± 5.32	
56–64	129 (25.96)	11.84 ± 0.46		35.98 ± 2.51		57.9 ± 4.68	
≥65	255 (51.31)	11.78 ± 0.68		36.03 ± 2.68		58.27 ± 5.18	
Residence			0.782		<0.001		0.301
Non-urban	235 (47.28)	11.79 ± 0.63		35.64 ± 2.65		58.58 ± 5.20	
Urban	262 (52.72)	11.79 ± 0.63		36.44 ± 2.40		57.93 ± 4.96	
Education			0.160		<0.001		0.091
Primary school and below	165 (33.2)	11.82 ± 0.51		35.44 ± 2.62		57.69 ± 4.73	
Junior High School	135 (27.16)	11.71 ± 0.76		36.01 ± 2.58		58.83 ± 5.23	
High school/Vocational school	138 (27.77)	11.87 ± 0.48		36.6 ± 2.25		58.43 ± 4.71	
College/University and above	59 (11.87)	11.68 ± 0.84		36.69 ± 2.6		57.95 ± 6.32	
Employment			0.695		0.001		0.801
Employed	42 (8.45)	11.88 ± 0.40		36.88 ± 1.97		57.93 ± 4.69	
Retired	204 (41.05)	11.77 ± 0.66		36.33 ± 2.66		58.16 ± 5.22	
Other^a^	251 (50.5)	11.78 ± 0.63		35.71 ± 2.50		58.35 ± 5.04	
Monthly income *per capita*, CNY			0.478		<0.001		0.610
<2,000	129 (25.96)	11.79 ± 0.60		35.09 ± 2.63		58.01 ± 4.85	
2,000–5,000	223 (44.87)	11.75 ± 0.71		36.06 ± 2.67		58.56 ± 5.44	
>5,000	145 (29.18)	11.85 ± 0.51		36.94 ± 1.91		57.94 ± 4.7	
Marital status			0.836		0.914		0.025
Married	481 (96.78)	11.79 ± 0.63		36.08 ± 2.49		58.12 ± 5.00	
Other^b^	16 (3.22)	11.88 ± 0.34		35.69 ± 4.18		61.81 ± 6.36	
Medical insurance							
Urban Employee Basic Medical Insurance	245 (49.3)	11.8 ± 0.63	0.544	36.51 ± 2.44	<0.001	58.02 ± 5.12	0.569
New Rural Cooperative Medical Insurance	199 (40.04)	11.8 ± 0.62	0.730	35.48 ± 2.67	<0.001	58.67 ± 5.11	0.199
Urban Resident Basic Medical Insurance	45 (9.05)	11.73 ± 0.62	0.221	36.29 ± 2.33	0.767	57.82 ± 5.02	0.472
Retired Cadres Medical Insurance	2 (0.4)	12.00 ± 0.00	0.574	33.5 ± 0.71	0.089	56.5 ± 10.61	0.806
Commercial Medical Insurance	8 (1.61)	11.63 ± 0.74	0.336	37.25 ± 2.05	0.192	58.13 ± 4.12	0.991
No Insurance	3 (0.6)	12.00 ± 0.00	0.491	35.67 ± 0.58	0.580	55 ± 2.65	0.226
Duration of Illness, months			0.516		0.021		0.143
<1	344 (69.22)	11.78 ± 0.65		36.27 ± 2.50		58.60 ± 5.05	
1–6	79 (15.9)	11.85 ± 0.51		35.61 ± 2.84		57.66 ± 4.97	
>6	74 (14.89)	11.74 ± 0.64		35.61 ± 2.39		57.16 ± 5.22	

^a^Includes unemployment, housewives, self-employed persons, and others. ^b^Includes unmarried, divorced, and widowed.

### Knowledge, attitudes and practice dimensions

The mean score of knowledge, attitude and practice were 11.79 ± 0.63 (possible range: 0–12), 36.06 ± 2.55 (possible range: 12–60), and 58.24 ± 5.08 (possible range: 14–70), respectively. Male participants demonstrated a greater tendency to hold constructive views and showed higher intent to commit to rehabilitation. Residence was significantly linked to attitude (*p* < 0.001), with urban residents more likely to exhibit positive attitudes. Education level was significance for both attitude (*p* < 0.001) and practice (*p* = 0.001), with higher education levels correlating to a higher likelihood of positive attitudes and practice. Monthly income per capita was significant for attitude (*p* < 0.001), indicating a greater likelihood of positive attitudes with higher income. Employment status was associated with practice (*p* = 0.001), suggesting that employed individuals were more likely to adopt favorable practice. Marital status demonstrated significance concerning practice (*p* = 0.025), with those having other marital statuses being more likely to engage in positive practice (Table 1).

Overall, participants demonstrated a high level of knowledge regarding stroke, with the mean score approaching the maximum possible (11.79 ± 0.63; range 0–12). While most participants were well-informed about common risk factors and general effects of stroke, certain misconceptions persisted, particularly regarding the appropriate time window for thrombolysis and the importance of early recovery-oriented training (Supplementary Table 1).

A notable majority, approximately 71.83%, expressed strong enthusiasm for participating in rehabilitation programs (A2). A high 94.37% believed in the importance of rehabilitation and were interested in learning more (A7). Also, 94.16% felt that rehabilitation could significantly improve daily life (A8). Yet, 55.94% were unwilling to bear rehabilitation costs (A9) (Supplementary Table 2).

The degree of interest in various rehabilitative activities varied considerably among respondents. A majority expressed readiness for inpatient rehabilitation (94.97%), regular health education (95.98%), and balance training such as Tai Chi (95.77%). However, lower levels of practice were observed for spasm management (54.73%), motor function assessment (41.65%), motor rehabilitation training (42.45%), and antidepressant treatment (42.05%). Swallowing function screening had moderate acceptance, with 75.86% willing to participate (Supplementary Table 3).

### Univariate and multivariate logistic regression analyses

The results of univariate and multivariate logistic regression analyses for factors associated with practice scores. In the univariate analysis, higher knowledge scores (OR: 1.595, 95% CI: 1.183–2.151, *p* = 0.002) and positive attitude scores (OR: 1.441, 95% CI: 1.313–1.582, *p* < 0.001) were significantly associated with better practice. Gender also emerged as a significant factor, with females being less likely to engage in favorable practice compared to males (OR: 0.564, 95% CI: 0.38–0.838, *p* = 0.005). In the multivariate analysis, while knowledge scores became non-significant (OR: 1.238, 95% CI: 0.874–1.753, *p* = 0.231), attitude scores remained a strong predictor of practice scores (OR: 1.438, 95% CI: 1.302–1.587, *p* < 0.001). Education level also demonstrated a significant association, with participants who had completed junior high school being more likely to exhibit favorable practice (OR: 1.932, 95% CI: 1.096–3.408, *p* = 0.023) compared to those with primary school education or below. Interestingly, other demographic factors, including age, residence, employment status, and income, did not show significant associations in the adjusted model (Table 2).

**Table 2:** Univariate and multivariate logistic regression analysis.

Variables	Univariate logistic regression		Multivariate logistic regression	
	OR (95%CI)	*p*	OR (95%CI)	*p*
Knowledge score	1.595 (1.183–2.151)	0.002	1.238 (0.874,1.753)	0.231
Attitude score	1.441 (1.313–1.582)	<0.001	1.438 (1.302,1.587)	<0.001
Gender				
Male	REF		REF	
Female	0.564 (0.38–0.838)	0.005	0.684 (0.436,1.073)	0.099
Age, years				
<55	REF			
56–64	1.027 (0.607–1.739)	0.920		
≥65	1.316 (0.825–2.101)	0.249		
Residence				
Non-urban	REF			
Urban	0.945 (0.65–1.375)	0.768		
Education				
Primary school and below	REF		REF	
Junior High School	2.167 (1.314–3.573)	0.002	1.932 (1.096,3.408)	0.023
High school/Vocational school	1.602 (0.994–2.582)	0.053	1.011 (0.593,1.724)	0.968
College/University and above	1.269 (0.685–2.35)	0.450	0.745 (0.370,1.500)	0.410
Employment				
Employed	REF			
Retired	1.557 (0.785–3.088)	0.205		
Other	1.328 (0.68–2.593)	0.406		
Monthly income *per capita*, CNY				
<2,000	REF			
2,000–5,000	1.269 (0.805–1.999)	0.305		
>5,000	1.317 (0.798–2.174)	0.282		
Marital status				
Married	REF			
Other	3.555 (0.798–15.83)	0.096		
Duration of illness				
<1 month	REF			
1–6 months	0.93 (0.555–1.559)	0.782		
>6 months	0.946 (0.556–1.611)	0.839		

### SEM

**Figure 1:**
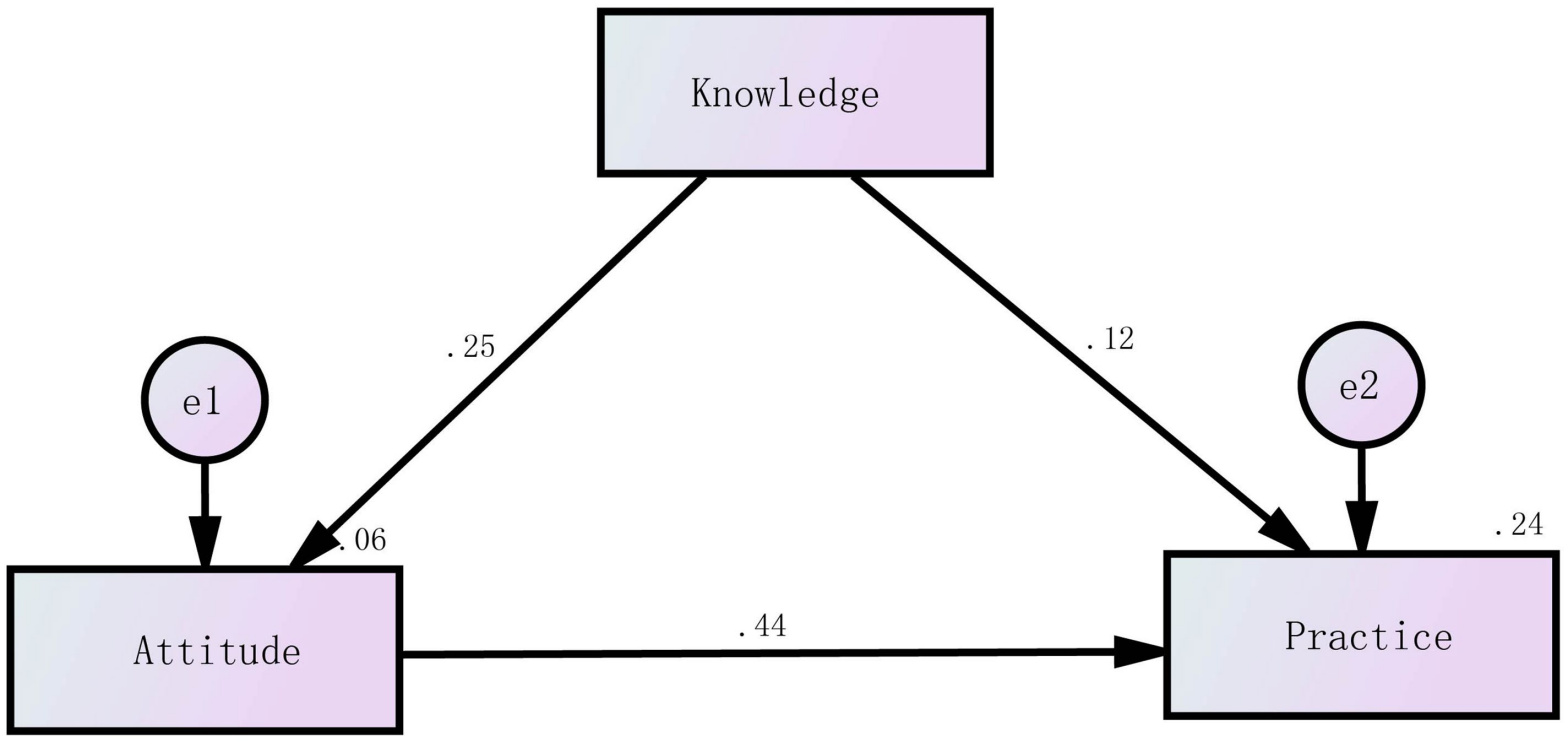
Structural equation model.

The SEM fitted well (Supplementary Table 4) and demonstrated that knowledge exhibits a positive and significant direct effect on attitudes (*β* = 0.249, *p* < 0.001), and attitudes had direct effects on practice (*β* = 0.443, *p* < 0.001). Knowledge had direct (*β* = 0.124, *p* = 0.002) and indirect effects (*β* = 0.111, *p* < 0.001) on practice (Figure 1; Table 3).

**Table 3:** Structural equation model.

Model paths		Total effects		Direct effect		Indirect effect	
		β (95%CI)	*p*	β (95%CI)	*p*	β (95%CI)	*p*
Attitude	Knowledge	0.249 (0.168, 0.330)	<0.001	0.249 (0.168, 0.330)	<0.001		
Practice	Knowledge	0.234 (0.152, 0.316)	<0.001	0.124 (0.045, 0.202)	0.002	0.111 (0.070, 0.151)	<0.001
	Attitude	0.443 (0.372, 0.515)	<0.001	0.443 (0.372, 0.515)	<0.001		

## Discussion

Stroke patients demonstrated sufficient knowledge, an unfavorable attitude and positive practice toward stroke and rehabilitation training. Healthcare providers play a crucial role in fostering positive attitudes through effective counseling strategies.

The study identified key barriers to patient engagement in rehabilitation, with financial constraints emerging as a significant factor influencing participation decisions. Although participants generally exhibited solid awareness of stroke risks and complications, pockets of misunderstanding still persisted, highlighting opportunities for targeted educational interventions (21, 22). Addressing financial concerns requires comprehensive strategies, such as improving insurance coverage and exploring innovative funding models, to enhance accessibility and adherence to rehabilitation programs (23, 24).

The significance of socioeconomic factors in influencing post-stroke attitudes and practice is clearly demonstrated in the study’s findings. Notably, educational attainment, particularly completion of junior high school, emerged as a significant predictor of practice, with these individuals being nearly twice as likely to engage in beneficial practice compared to those with primary education or below (25, 26). This correlation extends to monthly income, where higher income levels are associated with more positive attitudes (27). These results underscore the imperative for tailoring educational interventions and post-stroke support strategies to align with socioeconomic indicators. While advancements in medical standards have contributed to a decrease in stroke fatality rates, our findings highlight that many patients continue to face challenges with moderate to severe functional impairments, which significantly hinder their ability to live independently. Additionally, the study revealed that gaps in disease-related knowledge and negative attitudes among patients remain prevalent, potentially contributing to low engagement in rehabilitation programs (28–30).

The study reveals a coherent framework where correlations between knowledge, attitude, and practice contribute to a comprehensive understanding. Notably, patients possessing enhanced knowledge tend to exhibit more positive attitudes and a proclivity for beneficial practice. This observation aligns with the study (31). The implications underscore the pressing requirement for tailored psychological support and education interventions (32, 33). This alignment highlights the symbiotic relationship between attitude and practice, accentuating the pivotal role of addressing patients’ beliefs and viewpoints in facilitating adherence to recommended protocols. The documented positive correlations not only underscore the intrinsic interdependence of these factors but also corroborate established health behavior theories. This resonance underscores the potential of meticulously tailored educational interventions, serving not only to enrich patient comprehension but also to catalyze the cultivation of favorable attitudes and practice (15, 34, 35). Notably, although knowledge was significantly associated with practice in the univariate analysis, this relationship lost significance in the multivariate model, suggesting that the effect of knowledge might be primarily mediated through attitudes rather than directly influencing practice.

The SEM analysis showed that knowledge positively influenced attitudes, demonstrating that accurate information helps shape patients’ perceptions (36). Moreover, the observed positive impact of attitudes on practice highlights the importance of fostering constructive attitudes to effectively translate them into actionable behaviors during rehabilitation. Of particular note is the indirect effect, where knowledge’s influence on practice is channeled through attitudes. This mediation underscores the importance of enhancing patients’ attitudes, as they serve as a critical pathway through which knowledge positively influences practice (37–39).

This study had limitations. Firstly, its cross-sectional design limits the establishment of causal relationships among variables. Secondly, the study focused exclusively on patients from a single hospital, potentially limiting the generalizability of findings to a broader population. Additionally, the notably high knowledge scores (mean 11.79 out of 12) raise concerns of a ceiling effect, which may have implications for the interpretation of our SEM findings. We discuss this from three perspectives: the characteristics of the participant population, the design of the questionnaire, and the clinical context of stroke education. First, participants were recruited from a tertiary hospital setting where systematic stroke-related health education is routinely provided to inpatients to enhance treatment adherence and satisfaction, which likely elevated knowledge scores. Second, the dichotomous true/false format limited score variability and may have inflated correct responses. Third, all participants were stroke survivors with recent clinical exposure, which may have further increased disease-related awareness. The ceiling effect may reduce the variability required to detect strong associations, possibly underestimating the true effect of knowledge on practice in the SEM model. However, this near-maximal score distribution suggests a potential ceiling effect, which may reduce the ability to detect the true strength of associations involving knowledge in the structural equation model. The limited variance in knowledge scores could attenuate the observed relationships, particularly its direct effect on practice. These factors should be considered when interpreting the knowledge scores, and future studies might benefit from employing more diverse assessment methods. Lastly, reliance on self-reported data via a web-based questionnaire might introduce response bias and compromise the accuracy of information collected. The high knowledge scores observed may reflect the effects of systematic inpatient education in tertiary hospitals, potentially introducing a ceiling effect that limits generalizability and reduces the sensitivity of knowledge-related associations.

## Conclusion

Stroke patients demonstrated high knowledge levels, unfavorable attitudes, and positive practice toward rehabilitation. Knowledge positively influenced practice, mediated by attitudes. Socioeconomic factors, such as education and income, significantly shaped attitudes and practice. Customized strategies that tackle emotional well-being, economic constraints, and informational gaps are critical for fostering greater rehabilitation involvement.

## Data Availability

The original contributions presented in the study are included in the article/Supplementary material, further inquiries can be directed to the corresponding author.

## Footnotes

^1^ https://www.wjx.cn/
