# Supplementary material for: Patients’ knowledge, attitude, and practice toward stroke rehabilitation: a web-based cross-sectional study
Source: Front Public Health. 2025 Aug 7;13:1593802. doi: 10.3389/fpubh.2025.1593802 (PMC12367798; doi:10.3389/fpubh.2025.1593802)
Supplement: Supplementary file 1 [file Table_1.docx]

**Supplementary Table 1. Knowledge**

| Statement | Correct | Incorrect |
| --- | --- | --- |
| 1. Rapid stroke identification can be facilitated using "Stroke 120": 1- Look for facial asymmetry; mouth corner deviation. 2- Check for arm weakness by raising both arms parallelly; unilateral weakness. 0- (Listen) Assess speech clarity and communication difficulties. | 496(99.8) | 1(0.2) |
| 2. The time window for intravenous thrombolysis treatment is within 24 hours after onset. (False) | 473(95.17) | 24(4.83) |
| 3. Common risk factors for stroke include hypertension, diabetes, smoking, and abnormal blood lipid levels. | 492(98.99) | 5(1.01) |
| 4. Stroke typically leads to varying degrees of functional impairments, encompassing sensory and motor deficits, speech or communication impairments, cognitive dysfunction, emotional and psychological disturbances, and swallowing difficulties. | 495(99.6) | 2(0.4) |
| 5. Long-term oral anticoagulant drugs are not necessary after ischemic stroke. (False) | 470(94.57) | 27(5.43) |
| 6. Chronic patients requiring long-term treatment should be followed up once within 2 to 4 weeks after discharge, and subsequently at 3, 6 months, and 1 year intervals. | 496(99.8) | 1(0.2) |
| 7. Post-discharge, regular monitoring of blood pressure, reevaluation of blood glucose, lipid levels, electrocardiogram, cervical vascular ultrasound, brain CT, MRI, etc., are essential to assess stroke risk and adjust the treatment plan based on the examination results. | 494(99.4) | 3(0.6) |
| 8. Dietary habits need not be overly strict after discharge, but reducing smoking and alcohol consumption is important. (False) | 477(95.98) | 20(4.02) |
| 9. Rehabilitation, prevention, and acute-phase treatment of stroke hold equal significance. | 497(100) | 0(0) |
| 10. Effective rehabilitation treatment plays a crucial role in restoring a healthy lifestyle and reducing disease burden. | 491(98.79) | 6(1.21) |
| 11. Even after stabilizing, early recovery-oriented training is not advisable. (False) | 482(96.98) | 15(3.02) |
| 12. Muscle weakness and spasms significantly influence the recovery of motor function. | 496(99.8) | 1(0.2) |

**Supplementary Table 2. Attitudes**

| Attitude | | Very positive | Positive | Neutral | Negative | Very negative |
| --- | --- | --- | --- | --- | --- | --- |
| 1. The functional impairments following a stroke encompass motor, speech, cognitive, sensory, swallowing, and psychological aspects. Please indicate your level of anxiety regarding the following impairments: | |  |  |  |  |  |
| 1.1 Motor Function Impairment. | | 0(0) | 5(1.01) | 205(41.25) | 101(20.32) | 186(37.42) |
| 1.2 Sensory Function Impairment. | | 2(0.4) | 3(0.6) | 62(12.47) | 174(35.01) | 256(51.51) |
| 1.3 Speech and Language Impairment. | | 1(0.2) | 6(1.21) | 357(71.83) | 47(9.46) | 86(17.3) |
| 1.4 Swallowing Impairment. | | 2(0.4) | 10(2.01) | 393(79.07) | 68(13.68) | 24(4.83) |
| 1.5 Emotional Disturbance. | | (0) | 8(1.61) | 205(41.25) | 262(52.72) | 22(4.43) |
|  | |  |  |  |  |  |
| 2. I believe that rehabilitation therapy is of paramount importance and I am interested in learning more about related knowledge. | | 0(0) | 2(0.4) | 5(1.01) | 21(4.23) | 469(94.37) |
| 3. I believe that rehabilitation training can significantly improve daily life. | | 0(0) | 0(0) | 0(0) | 29(5.84) | 468(94.16) |
| 4. I believe that rehabilitation should commence as early as possible. | | 0(0) | 1(0.2) | 1(0.2) | 13(2.62) | 482(96.98) |
| 5. I believe that it is beneficial to appropriately increase training intensity if conditions allow. | 1(0.2) | | 2(0.4) | 4(0.8) | 13(2.62) | 477(95.98) |
| 6. I am concerned that even with active rehabilitation training, complete restoration to normal might not be achieved. | 17(3.42) | | 26(5.23) | 41(8.25) | 161(32.39) | 252(50.7) |
| 7. I wish to receive more guidance on rehabilitation training. | 423(85.11) | | 68(13.68) | 2(0.4) | 2(0.4) | 2(0.4) |
| 8. Rehabilitation training incurs costs, and I am unwilling to bear such expenses. | 44(8.85) | | 4(0.8) | 13(2.62) | 158(31.79) | 278(55.94) |

**Supplementary Table 3. practice**

| Practice | Always | Often | Sometimes | Rarely | Never |
| --- | --- | --- | --- | --- | --- |
| Regarding the following screening and rehabilitation methods, please indicate your willingness to undergo: |  |  |  |  |  |
| 1. Inpatient rehabilitation at comprehensive hospitals and rehabilitation centres during the recovery period. | 0(0) | 12(2.41) | 13(2.62) | 472(94.97) | 0(0) |
| 2. Motor Function Impairment |  |  |  |  |  |
| 2.1 Spasm Management. | 0(0) | 3(0.6) | 272(54.73) | 78(15.69) | 144(28.97) |
| 2.2 Motor Function Assessment. | 0(0) | 3(0.6) | 207(41.65) | 81(16.3) | 206(41.45) |
| 2.3 Motor Rehabilitation Training. | 0(0) | 1(0.2) | 211(42.45) | 82(16.5) | 203(40.85) |
| 2.4 Neuromuscular Electrical Stimulation. | 0(0) | 3(0.6) | 210(42.25) | 83(16.7) | 201(40.44) |
| 3. Sensory Function Impairment |  |  |  |  |  |
| 3.1 Sensory Impairment Assessment. | 0(0) | 1(0.2) | 64(12.88) | 155(31.19) | 277(55.73) |
| 3.2 Re-adaptation Training using various sensory stimuli. | 0(0) | 2(0.4) | 58(11.67) | 158(31.79) | 279(56.14) |
| 4. Swallowing Function Impairment |  |  |  |  |  |
| 4.1 Swallowing Function Screening. | 0(0) | 7(1.41) | 377(75.86) | 72(14.49) | 41(8.25) |
| 4.2 Swallowing Function Assessment. | 0(0) | 7(1.41) | 377(75.86) | 74(14.89) | 39(7.85) |
| 4.3 Oral Hygiene Management. | 0(0) | 3(0.6) | 188(37.83) | 250(50.3) | 56(11.27) |
| 5. Emotional Disturbances |  |  |  |  |  |
| 5.1 Psychological Evaluation. | 0(0) | 3(0.6) | 15(3.02) | 9(1.81) | 470(94.57) |
| 5.2 Antidepressant Treatment when necessary. | 1(0.2) | 2(0.4) | 209(42.05) | 230(46.28) | 55(11.07) |
| 6. Receive Health Education regularly. | 0(0) | 1(0.2) | 3(0.6) | 16(3.22) | 477(95.98) |
| 7. Engage in Balance Training (such as Tai Chi). | 0(0) | 0(0) | 7(1.41) | 14(2.82) | 476(95.77) |

**Supplementary Table 4.** Model fit.

| **Indicators** | **Reference** | **Results** |
| --- | --- | --- |
| RMSEA | <0.08 | 0.000 |
| SRMR | <0.08 | 0.000 |
| TLI | >0.80 | 1.000 |
| CFI | >0.80 | 1.000 |
